# 1719. Duration of antibodies after immunization with the quadrivalent influenza vaccine in children after hematopoietic stem cell transplantation or chemotherapy compared to healthy children

**DOI:** 10.1093/ofid/ofad500.1551

**Published:** 2023-11-27

**Authors:** Yoon Kyung Cho, Bin Ahn, Sujin Choi, Ye Ji Kim, Kyu Ri Kang, Hyunmi Kang, Jin Han Kang

**Affiliations:** The Catholic University of Korea, Seoul St. Mary's Hospital, Seoul, Seoul-t'ukpyolsi, Republic of Korea; Seoul National University Children's Hospital, Seoul, Seoul-t'ukpyolsi, Republic of Korea; The Catholic University of Korea, Seoul St. Mary's Hospital, Seoul, Seoul-t'ukpyolsi, Republic of Korea; The Catholic University of Korea, Seoul, Seoul-t'ukpyolsi, Republic of Korea; The Catholic University of Korea, Seoul St. Mary's Hospital, Seoul, Seoul-t'ukpyolsi, Republic of Korea; The Catholic University of Korea, Seoul St. Mary's Hospital, Seoul, Seoul-t'ukpyolsi, Republic of Korea; The Catholic University of Korea, Seoul, Seoul-t'ukpyolsi, Republic of Korea

## Abstract

**Background:**

Children that have undergone hematopoietic stem cell transplantation (HSCT) are at a higher risk for severe influenza. The purpose of this study was to compare the duration of antibodies after immunization with the quadrivalent influenza vaccine (QIV) in children after hematopoietic stem cell transplantation or chemotherapy compared to healthy children.

**Methods:**

This was a prospective study of children below 18 years old that received the QIV influenza vaccine during the 2021-2022 influenza vaccination season. Children were divided into 3 groups as follows: a) post-HSCT, b) post-chemotherapy, and c) immunocompetent children as a healthy control. Blood samples were taken immediately before immunization, at 1 month, 3 months, 6 months, and 12 months after immunization. Hemagglutination Inhibition (HI) assays were performed. Seropositive rate was defined by the percentage of patients with HI titer ≥1:40. Seroconversion rate (SCR) was defined as follows: 1) in patients with a pre-vaccination HI titer < 1:10, rise in post-vaccination HI titers ≥1:40, or in patients with pre-vaccination HI titers ≥1:40, a 4-fold increase.

**Results:**

A total of 60 children were included in the preliminary analyses, n=20 in each of the three groups. The SPR at post-vaccination 1 month for both A/H1N1 and H3N2 exceeded 80% in all three groups. The SPR remained above 60% at 3 months post-immunization for both A antigens. However, by 6 months post-vaccination, the SPR dropped to 50% in the post-chemotherapy group for the A/H1N1 antigen, and the geometric mean titers (GMTs) were 26.39. For B/Victoria and B/Yamagata, the SPR was below 40% in the post-HSCT and post chemotherapy group at 1 months and below 20% by 3 months. The GMTs were also below 1:40 for both antigens. In the normal control group, the SPR for B/Victoria was 50% at 1 months and 45% at 3 months post-immunization, and for B/Yamagata, 50% at 1 months and 55% at 3 months post-immunization. However, the GMTs were below 1:40 by 3 months post-immunization.

**Conclusion:**

For A/H1N1, the SPR and GMTs at 6 months in the post-chemotherapy was low. In both the post-HSCT and chemotherapy group, HI titers were low for both B antigens at 1-month post-vaccination, and GMTs showed < 1:40. B antigens contained within QIV need to illicit better protection against Influenza in children.

**Disclosures:**

**All Authors**: No reported disclosures

